# Supplementary material for: Decorin-mediated suppression of tumorigenesis, invasion, and metastasis in inflammatory breast cancer
Source: Commun Biol. 2021 Jan 15;4:72. doi: 10.1038/s42003-020-01590-0 (PMC7811004; doi:10.1038/s42003-020-01590-0)

Supplementary Information for

Decorin-mediated suppression of tumorigenesis, invasion, and metastasis in inflammatory breast cancer

Authors: Xiaoding Hu,[1,2] Emilly S Villodre,[1,2] Richard Larson,[2,3] Omar M Rahal,[2,3] Xiaoping Wang,[1,2] Yun Gong [2,4] Juhee Song,[5] Savitri Krishnamurthy,[2,4] Naoto T. Ueno,[1,2] Debu Tripathy,[1,2] Wendy A Woodward,[2,3] and Bisrat G Debeb[1,2*]

This file includes:

Supplementary Figures 1-14

Supplementary Figure 15 contains all uncropped western blots for main and supplementary figures.

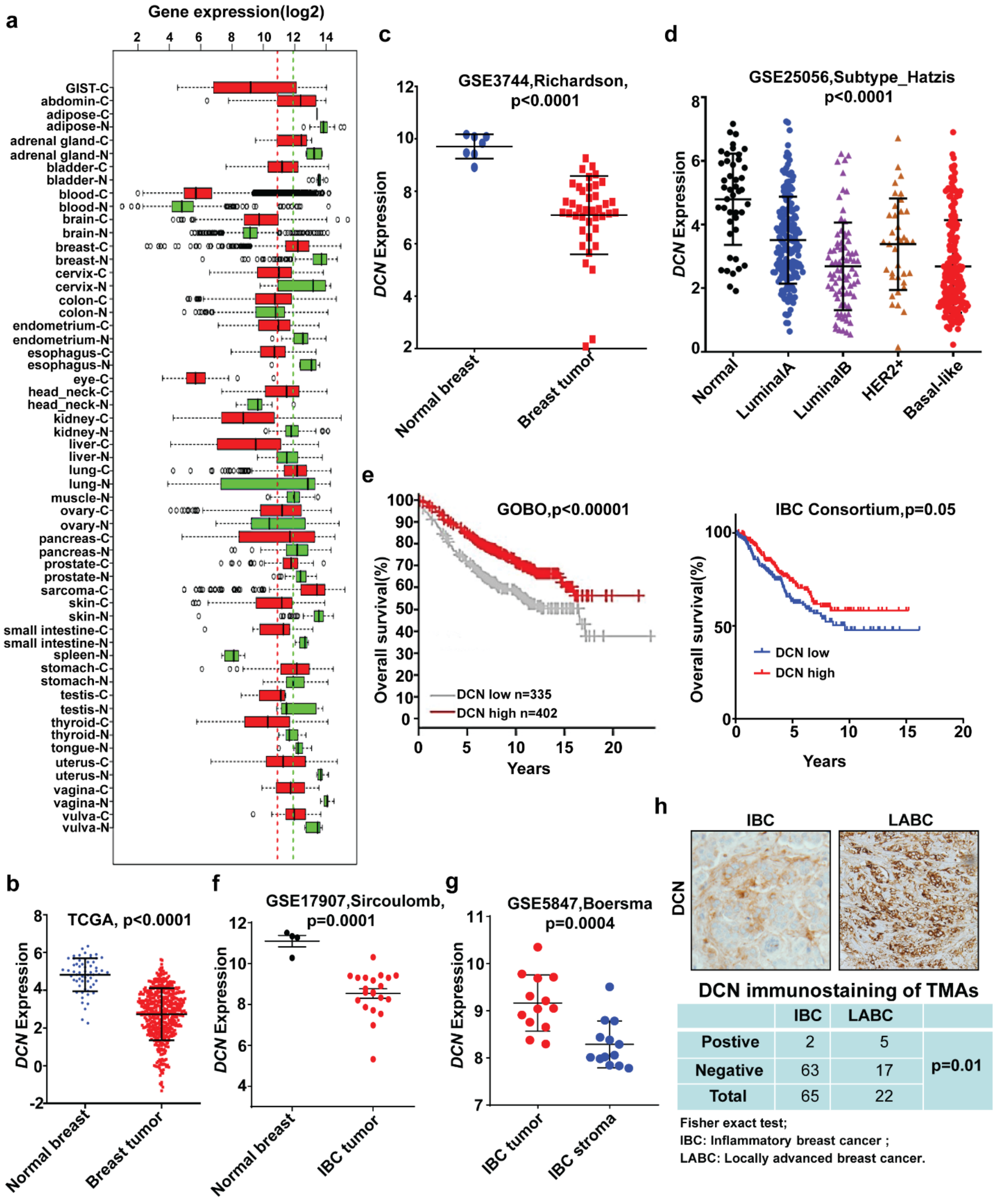

	IBC	LABC	
Postive	2	5	p=0.01
Negative	63	17	
Total	65	22	

Supplementary Figure 1: DCN is downregulated in aggressive breast tumors.

a, Decorin (*DCN*) mRNA expression pattern was analyzed in normal and tumor tissues across cancer types from the online database Gene Expression across Normal and Tumor tissues

(GENT), which contains more than 34,000 samples. N, normal; C, cancer. **b and c,** DCN is downregulated in breast tumors relative to normal tissues, as shown in The Cancer Genome Atlas (TCGA) **(b)** and Richardson dataset (GSE3744) **(c)**. **d,** DCN expression is downregulated in more aggressive, basal-like breast cancer subtypes (Hatzis dataset, GSE25066). **e,** High DCN expression is associated with better survival outcomes, as indicated by the Gene Expression Based Outcome (GOBO) and Inflammatory Breast Cancer (IBC) Consortium datasets. **f,** DCN expression is downregulated in IBC compared with normal breast tissues, as indicated by the Sircoulomb dataset (GSE17907). **g**, The mRNA expression of DCN is lower in IBC tumor stroma compared with IBC tumor cells, as indicated by the Boersma dataset (GSE5847); **h,** DCN is expressed less often in the more aggressive IBC tumors than in locally advanced non-IBC breast cancer (LABC).

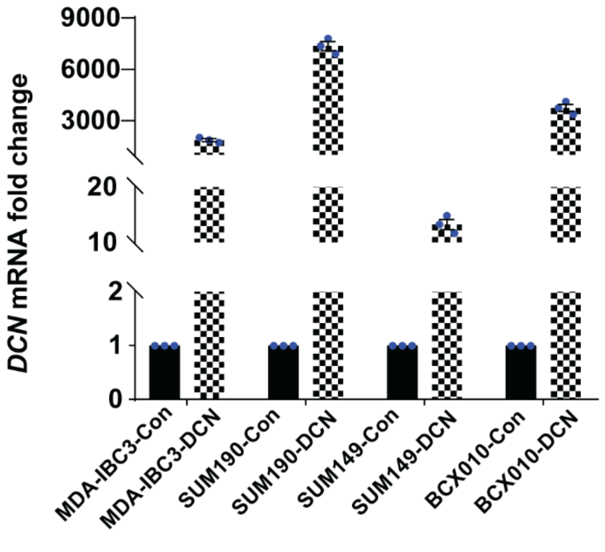

Supplementary Figure 2: Validation of *DCN* mRNA overexpression in IBC cell lines. *DCN* mRNA levels were assessed by quantitative RT-PCR in DCN-overexpressing and control IBC cell lines. Data are shown as mean ± s.e.m. Data are from three independent assays.

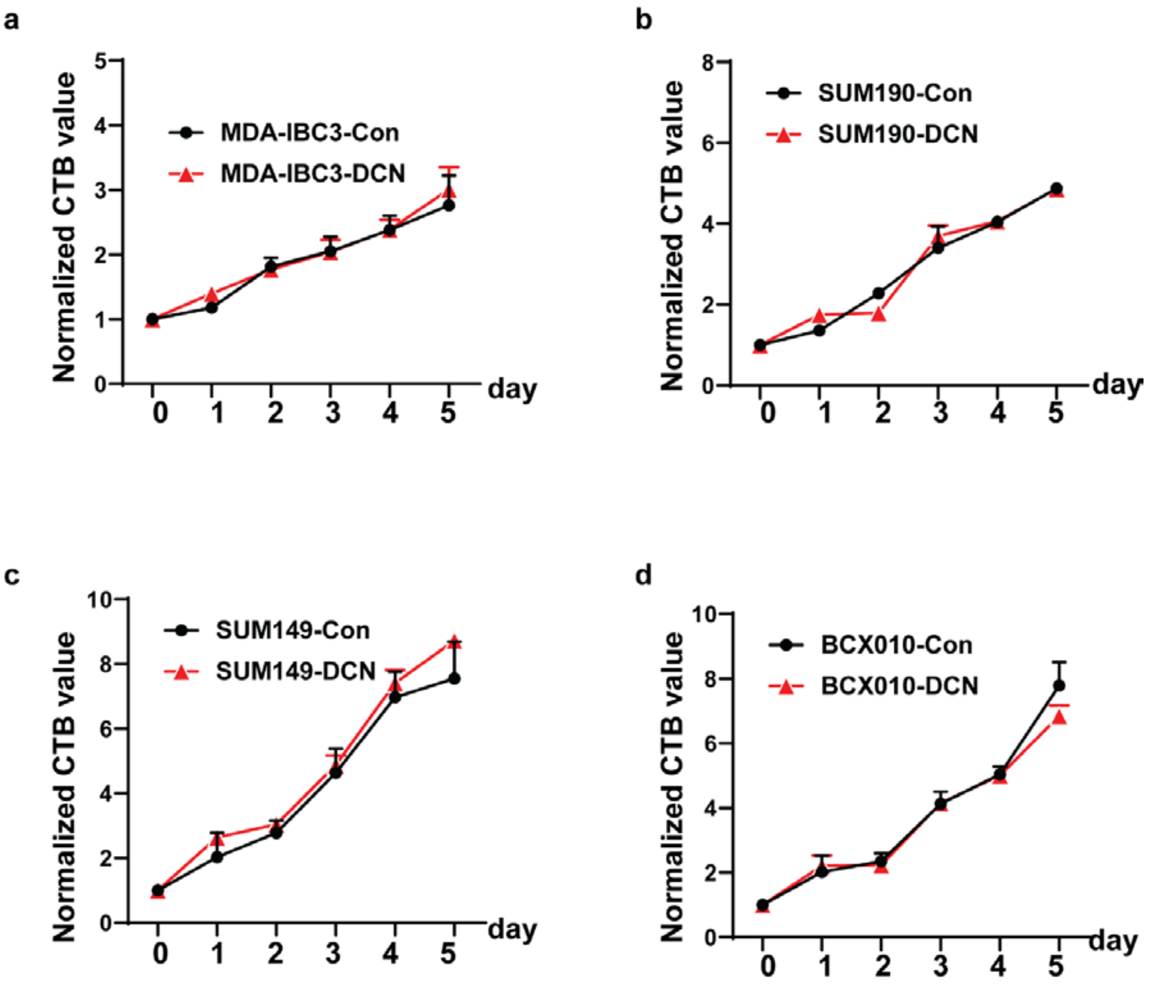

Supplementary Figure 3: DCN does not affect proliferation in IBC cell lines. Cell number was evaluated with a CellTiterBlue assay (Promega) on the indicated days. The fluorescence value of the cells measured at the start of the experiment was set as 1. Each cell line was evaluated separately at the indicated periods, and the fluorescence values of treated cells were normalized to their corresponding non-treated controls. a-d, Results in MDA-IBC3 cells (**a**), SUM149 cells (**b**), SUM190 cells (**c**), and BCX010 cells (**d**) show no significant differences between the DCN-overexpressing and control groups. Results were normalized to the controls. All data are represented as means ± s.e.m, with all experiments done in triplicate.

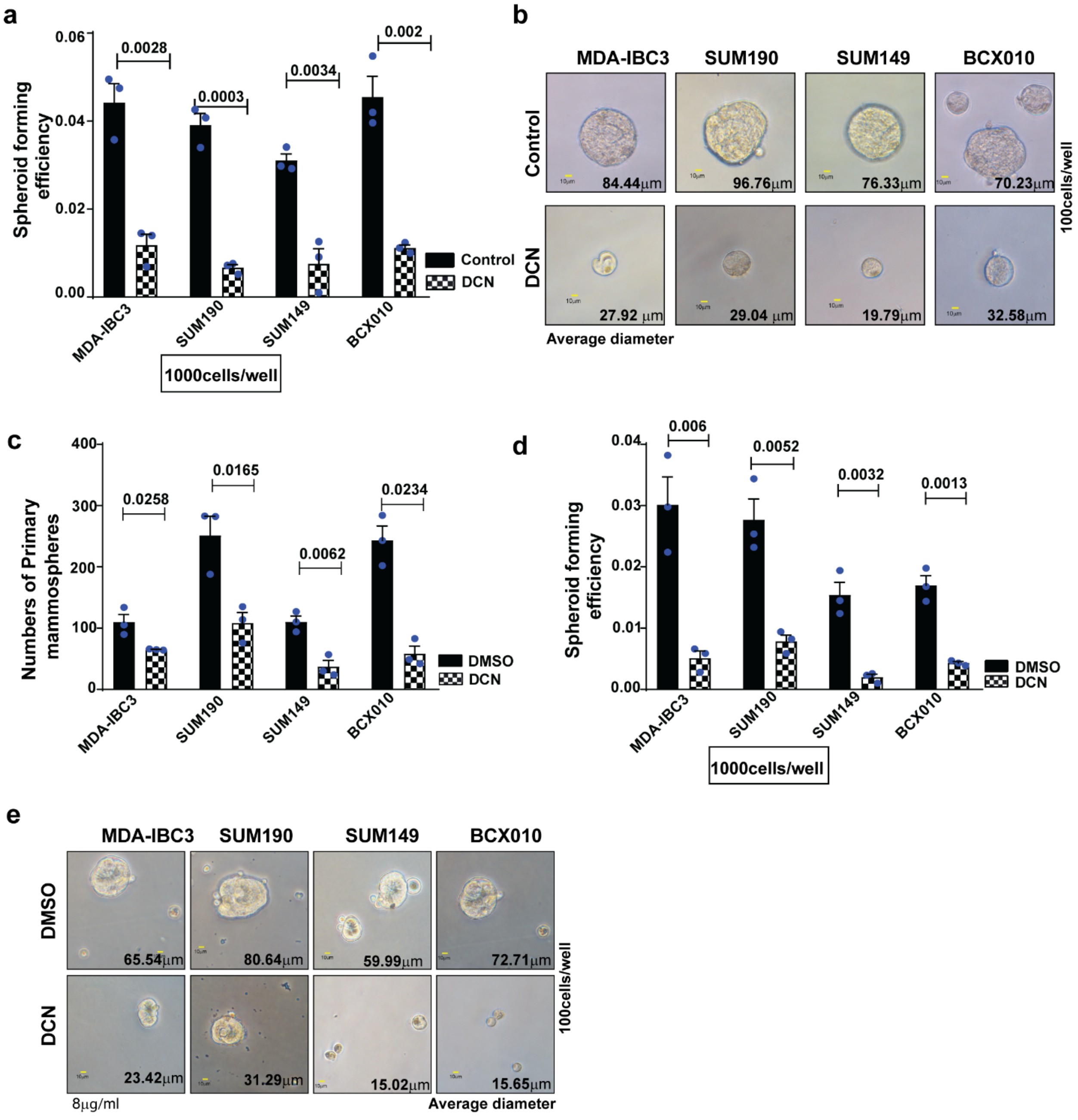

Supplementary Figure 4: DCN reduces spheroid-forming efficiency and spheroid size. (**a-b**), DCN overexpression in IBC cell lines reduces (**a**) spheroid forming efficiency (1000 cells plated per well) and (**b**) spheroid size; (**c-e**), Treatment of IBC cell lines with recombinant DCN protein (8 μg/mL) suppresses primary mammosphere formation (**c**), spheroid forming efficiency (1000 cells/well plated) (**d**), and (**e**) spheroid size (100 cells plated per well) P values are from

Student's unpaired t tests. Data are shown as mean ± s.e.m. Data are from three independent assays. Representative images of spheroids (Scale = 10μm) showing decreased average diameter in DCN-treated vs vehicle-treated IBC cells.

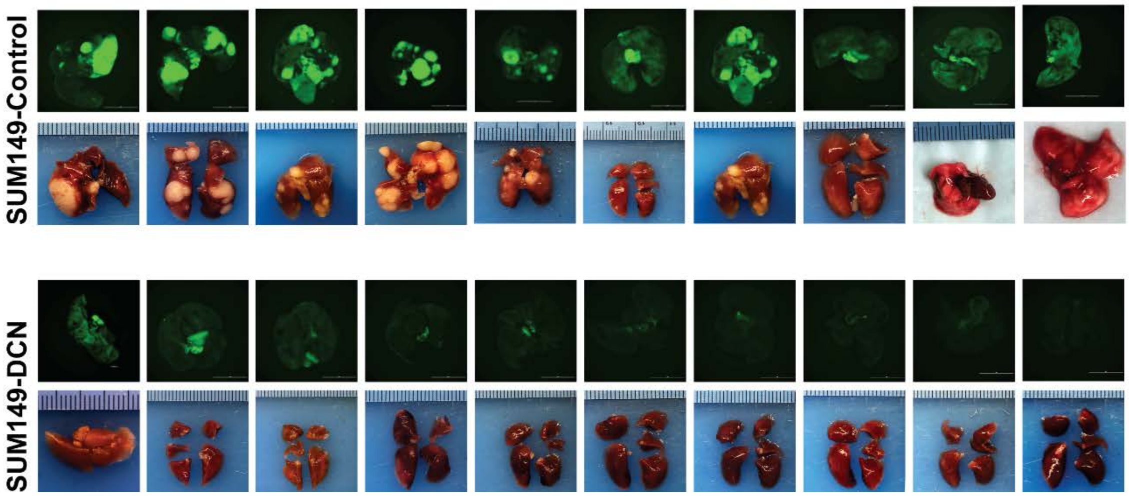

Supplementary Figure 5: DCN overexpression inhibits lung metastatic colonization in vivo

GFP-labeled DCN-overexpressing or control SUM149 cells were injected via tail vein into SCID/Beige mice (10 mice/group). Images of lungs from all of the mice in each group are shown here.

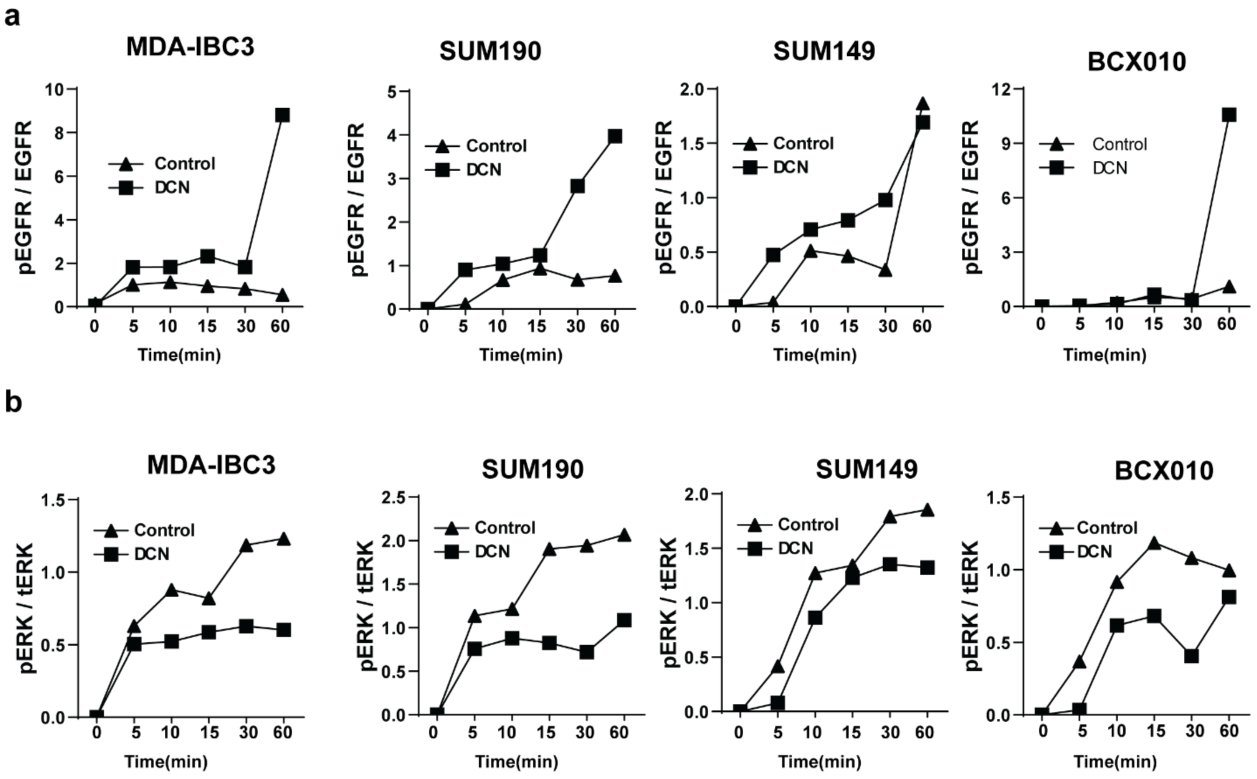

Supplementary Figure 6: p-EGFR/EGFR ratio (a) and p-ERK/t-ERK ratio (b) generated from Figure 3g.

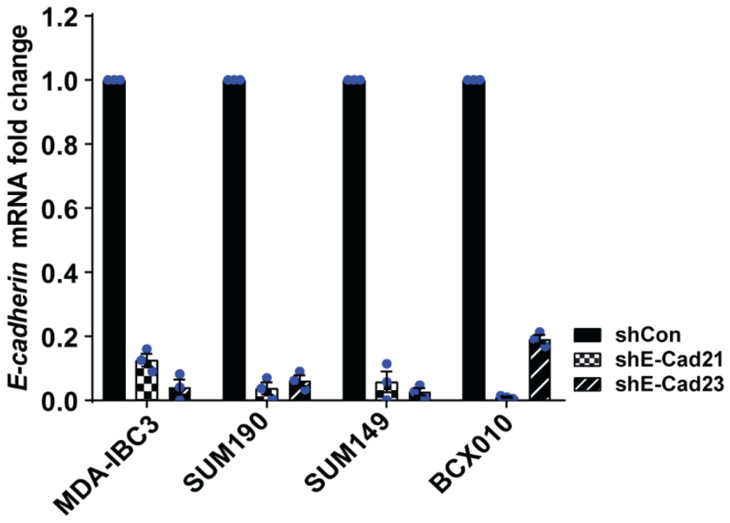

Supplementary Figure 7: Validation of *E-cadherin* mRNA knockdown in IBC cell lines. *E-cadherin* mRNA levels were measured by quantitative RT-PCR in four cell lines with E-cadherin knockdown. Data are shown as mean±s.e.m. Data are from three independent assays.

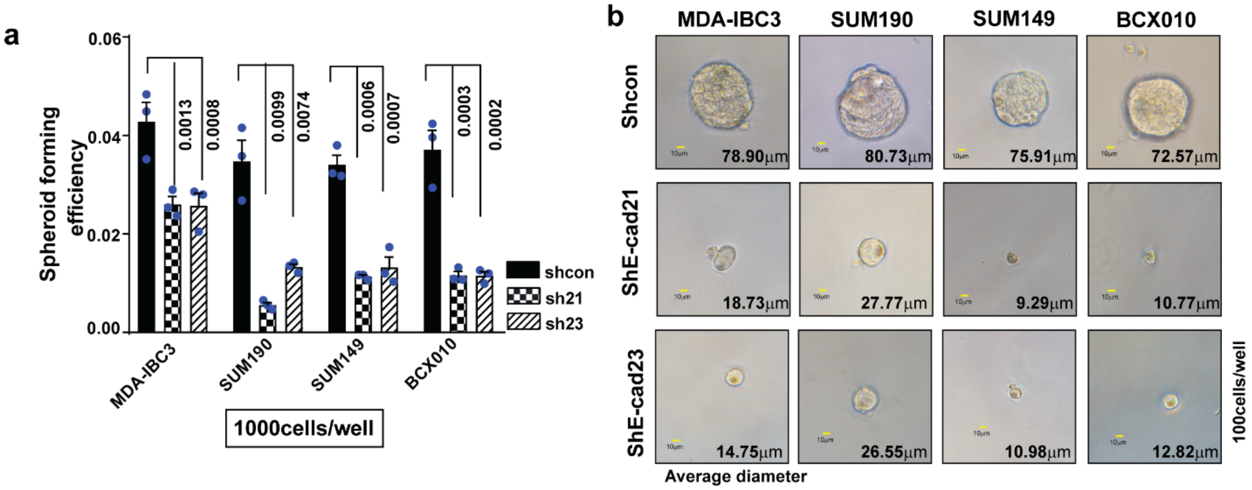

Supplementary Figure 8: E-cadherin knockdown in IBC cells reduces spheroid-formation efficiency and spheroid size. a, E-cadherin knockdown in reduces spheroid-forming efficiency (1000 cells plated per well); *P* values are from Student's unpaired *t* tests. **b**, E-cadherin knockdown reduces spheroid size in IBC cell lines. Representative images of spheroids (scale = 10 μm) indicates smaller average diameter of E-cadherin knockdown IBC cell spheroids compared with control-cell spheroids (100 cells/well plated). Data are shown as mean±s.e.m. P values from Student's unpaired t tests. Data are from three independent assays.

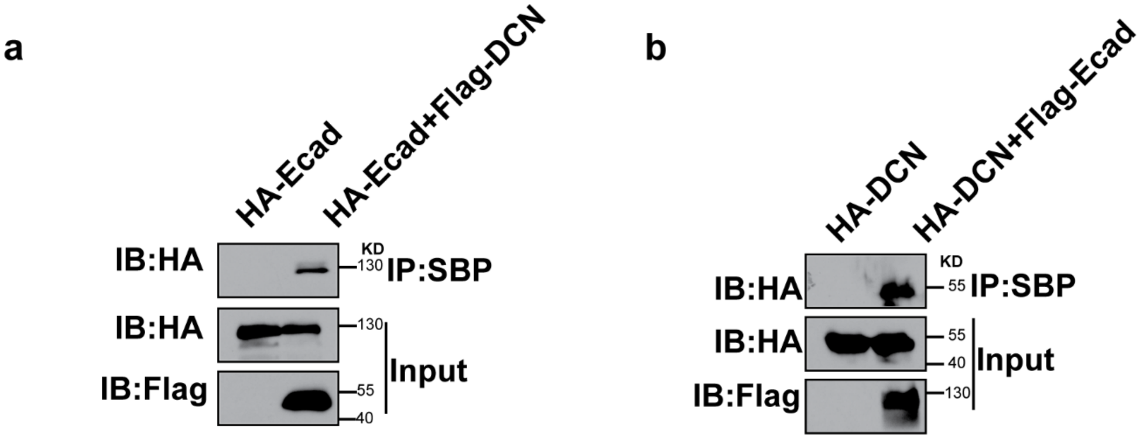

Supplementary Figure 9: DCN forms a complex with E-cadherin in HEK293T cells in vitro. a, HA-E-cadherin and Strep-E-cadherin-flag-DCN were co-transfected into 293T cells. **b**, HA-DCN and HA-DCN-flag-E-cadherin were co-transfected into HEK293T cells. Cell lysates were analyzed by immunoprecipitation and western blotting with anti-FLAG and anti-HA antibodies. Whole-cell lysates were blotted and shown as the input.

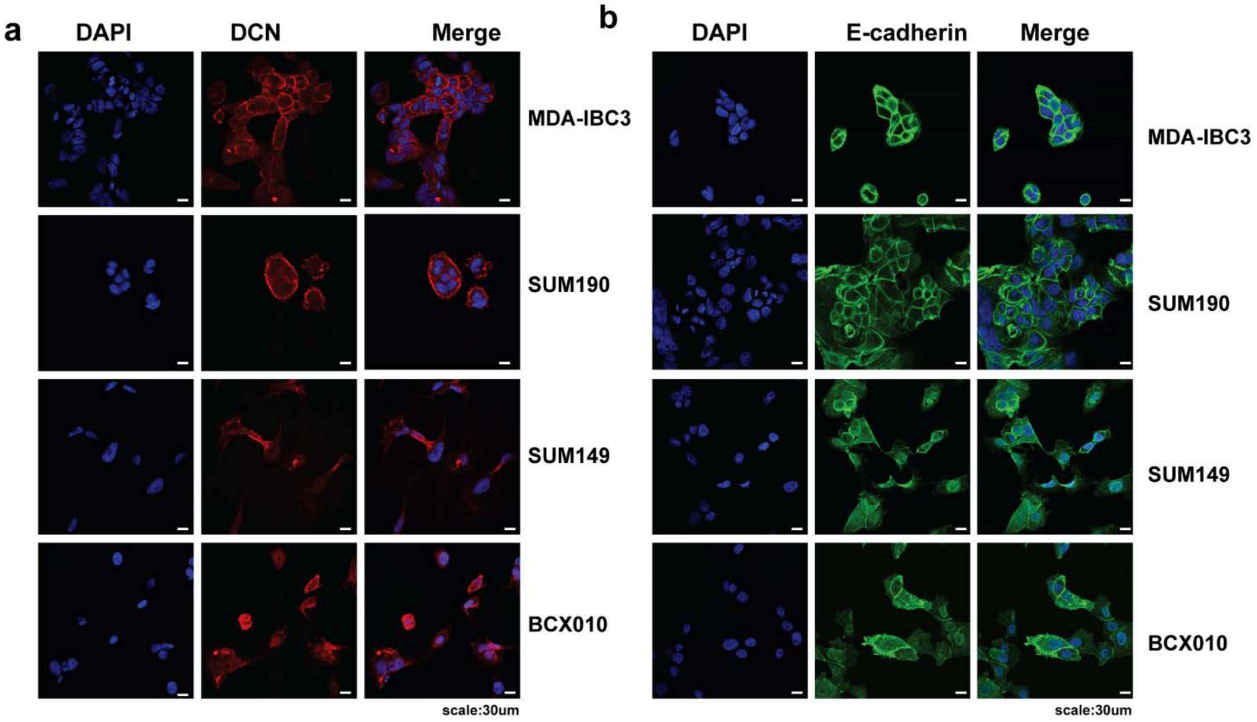

Supplementary Figure 10: DCN and E-cadherin are co-localized on the membrane.

Immunofluorescence staining of endogenous DCN and E-cadherin in IBC cell lines. **a and b,** Single-immunofluorescence staining shows localization of DCN (red) (**a**) and E-cadherin (green) (**b**) on the membranes of IBC cells. Scale bar: 30μm.

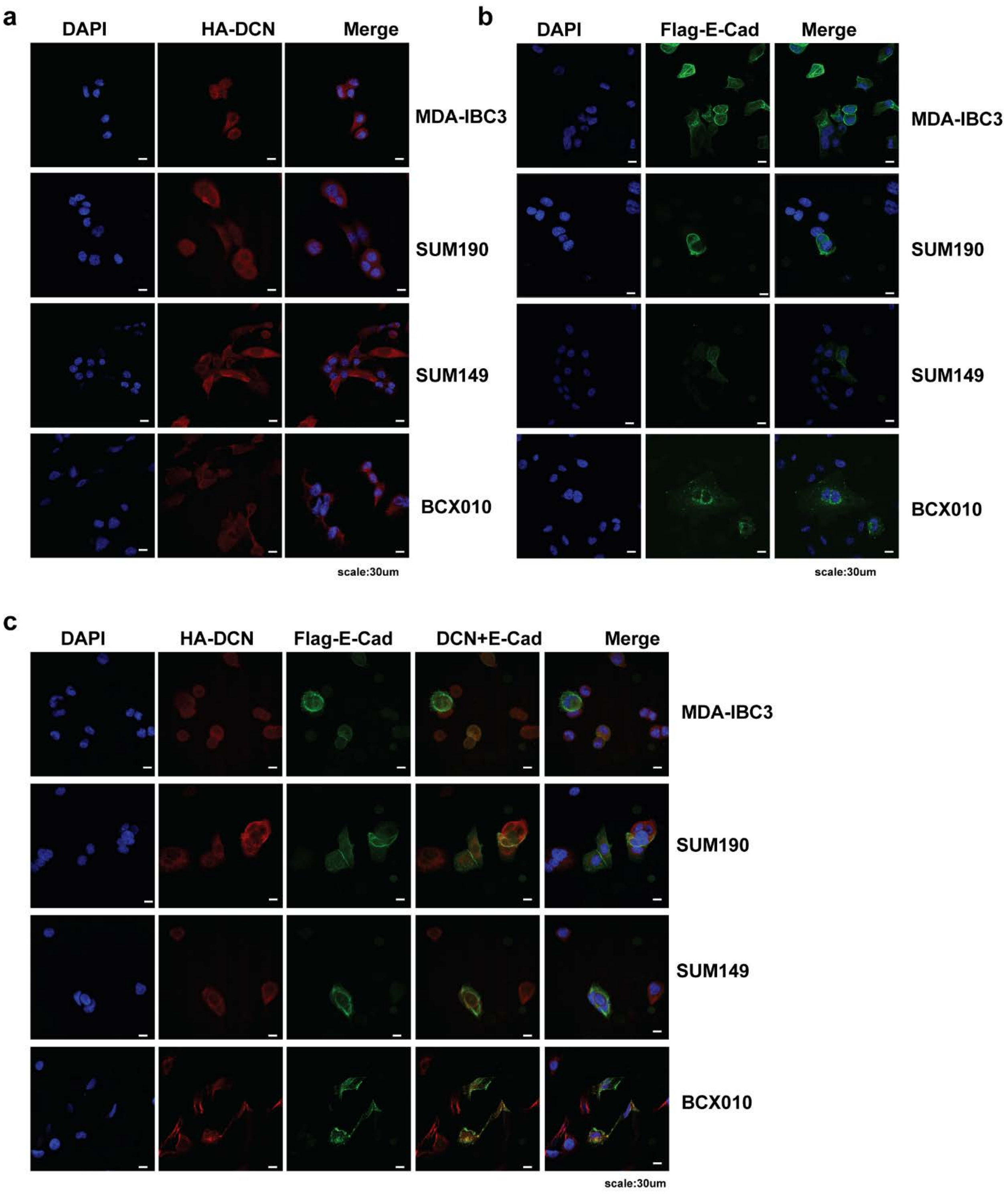

Supplementary Figure 11: HA-Decorin and Strep-flag-E-cadherin are co-localized on cell membranes. a and b, HA-DCN or strep-flag-E-cadherin was transfected individually into each of the indicated IBC cell lines, and subcellular localization was examined by using anti-HA

polyclonal antibody (red) or anti-Flag monoclonal antibody (green). Both were localized on the membranes of IBC cells. **c**, HA-DCN and Strep-flag-E-cadherin were co-transfected into IBC cells and the subcellular localization of both flags was examined by using an anti-HA polyclonal antibody (red) and anti-Flag monoclonal antibody (green). Both HA-DCN and Flag-E-cadherin were co-localized on the cell membranes. Scale bar: 30μm.

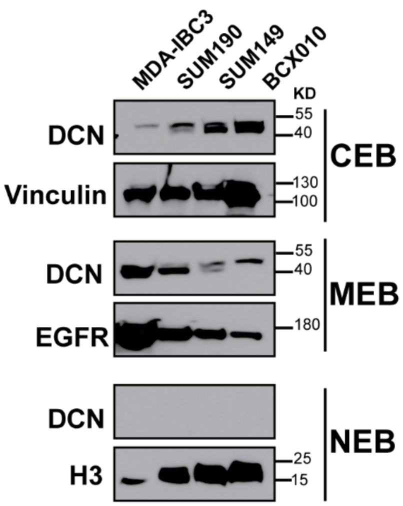

Supplementary Figure 12: DCN protein is localized in cytoplasm and membrane fractions in IBC cells. Cytoplasmic (CEB), membrane (MEB) and nuclear (NEB) fractions in IBC cell lines were separated and analyzed by immunoblotting. DCN is localized in the cytoplasm and membrane of these IBC cells. Vinculin, EGFR, and H3 served as markers for CEB, MEB and NEB, respectively.

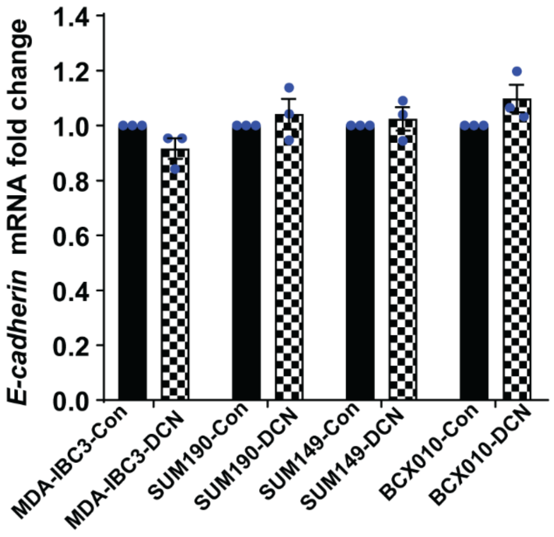

Supplementary Figure 13: DCN overexpression does not affect *E-cadherin* mRNA expression. *E-cadherin* mRNA levels were quantified by qRT-PCR in control and DCN-overexpressing IBC cell lines. Data are shown as mean ± s.e.m. P values from Student's unpaired t tests. Data are from three independent assays.

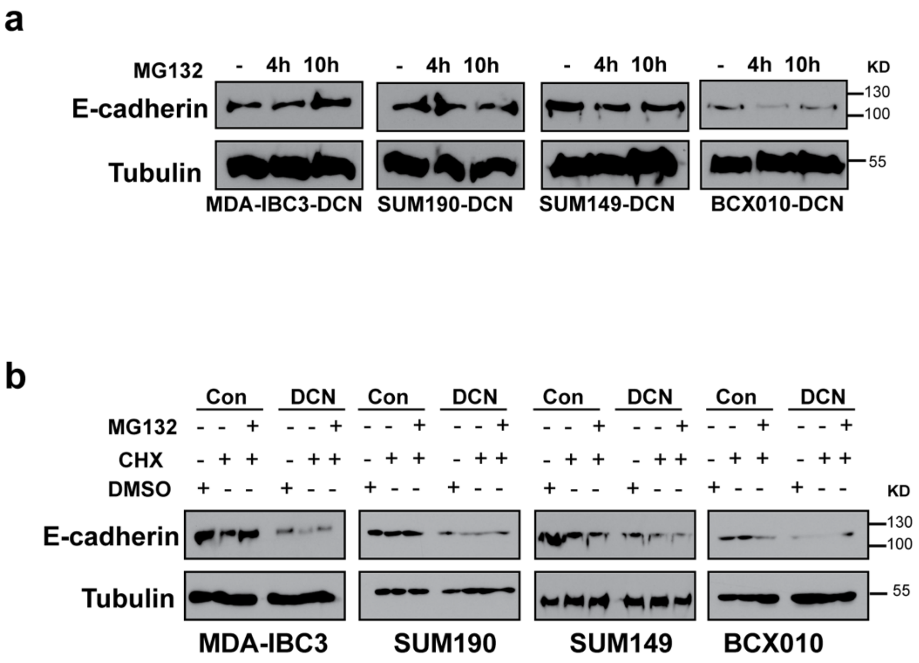

Supplementary Figure 14: Inhibiting proteasomes with MG132 treatment does not rescue E-cadherin expression in DCN-overexpressing IBC cells. a, DCN-overexpressing IBC cells treated with MG132 (50 nmol) for 4 hours or 10 hours showed no differences in E-cadherin protein levels over time. **b**, DCN-overexpressing and control IBC cells were treated with the protein synthesis inhibitor cycloheximide (CHX,100 μg/ml) with or without MG132 (50 nmol) for 10 hours. No differences in E-cadherin protein levels were noted between DCN-overexpressing and control cells. Collectively, these results indicate that DCN-mediated E-cadherin degradation is independent of proteasome activity.

Supplementary Figure 15. Uncropped western blots for main and supplementary figures as indicated.

Main figures: 1a, 3,4a and 4g, 5, 6

Supplementary figures: 9a and 9b, 12, 14a and 14b

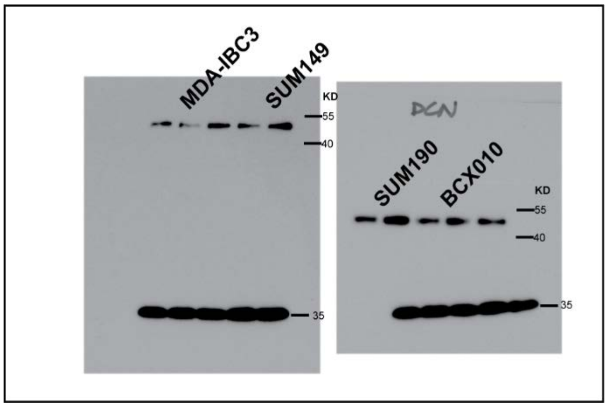

Fig1-a

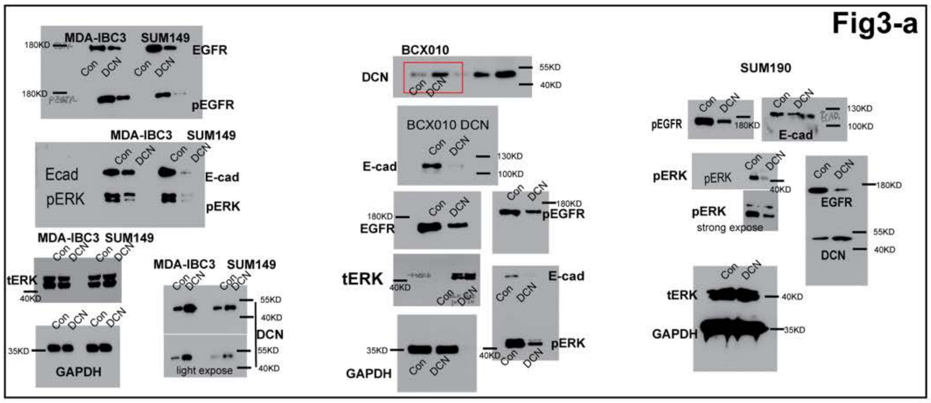
Fig3-a
MDA-IBC3
SUM149
EGFR
pEGFR
Con
DCN
180KD
E-cad
pERK
Ecad
tERK
40KD
GAPDH
35KD
55KD
light expose
BCX010
BCX010 DCN
130KD
100KD
SUM190
strong expose

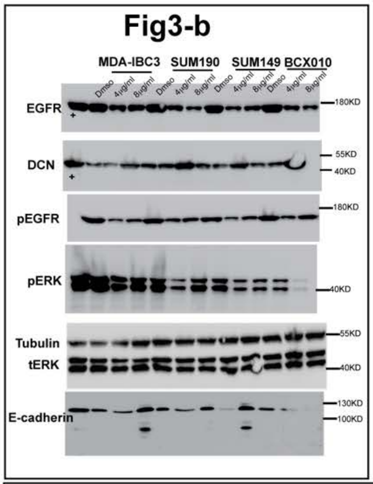
Fig3-b
MDA-IBC3
SUM190
SUM149
BCX010
EGFR
DCN
pEGFR
pERK
Tubulin
tERK
E-cadherin
180KD
55KD
40KD
130KD
100KD

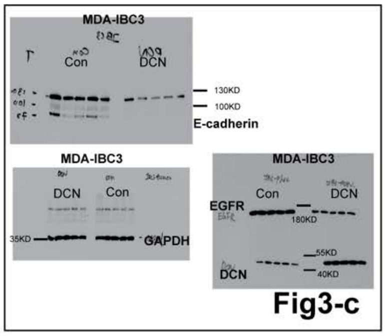
MDA-IBC3
Con
DCN
130KD
100KD
E-cadherin
GAPDH
35KD
EGFR
180KD
55KD
40KD
Fig3-c

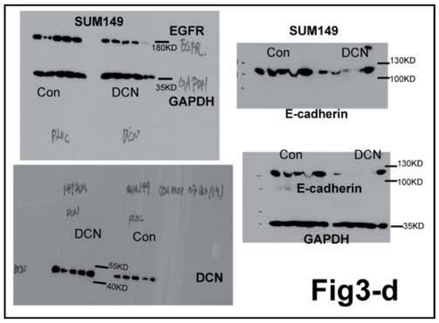
SUM149
EGFR
180KD
35KD
GAPDH
Con
DCN
130KD
100KD
E-cadherin
55KD
40KD
Fig3-d

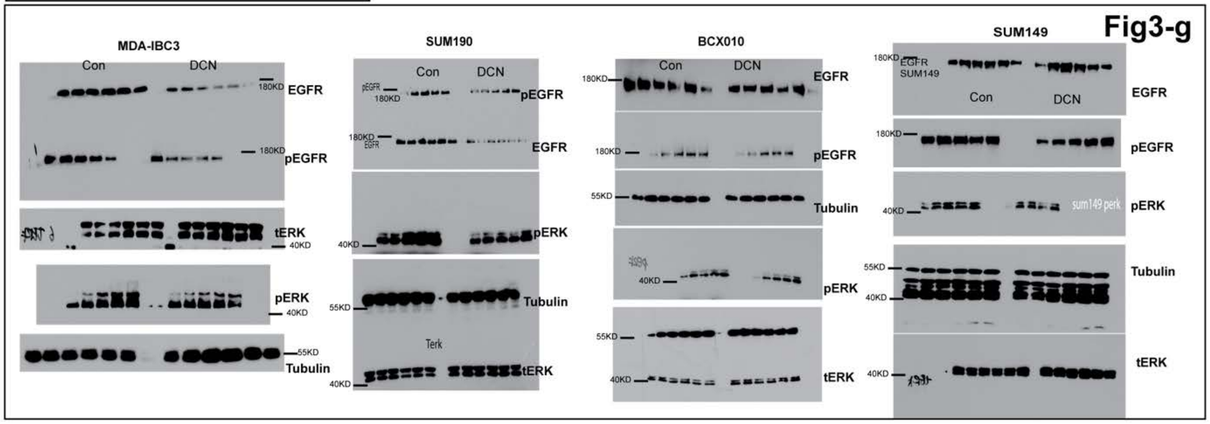
Fig3-g
MDA-IBC3
SUM190
BCX010
SUM149
Con
DCN
EGFR
pEGFR
tERK
pERK
Tubulin
180KD
55KD
40KD
Terk
sum149 perk

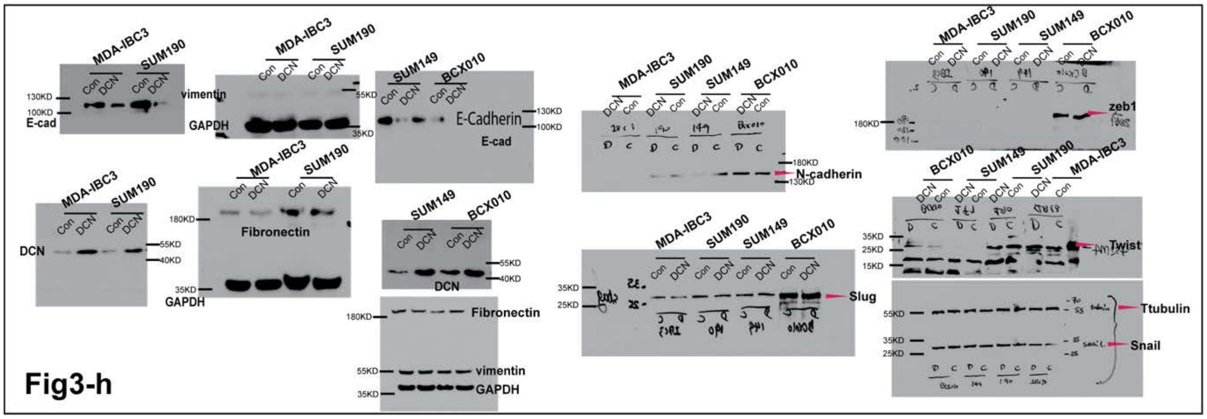
MDA-IBC3
SUM190
SUM149
BCX010
Con
DCN
130KD
100KD
E-cad
vimentin
GAPDH
55KD
35KD
E-Cadherin
N-cadherin
180KD
zeb1
DCN
40KD
Fibronectin
Twist
Slug
Ttubulin
Snail
25KD
15KD
Fig3-h

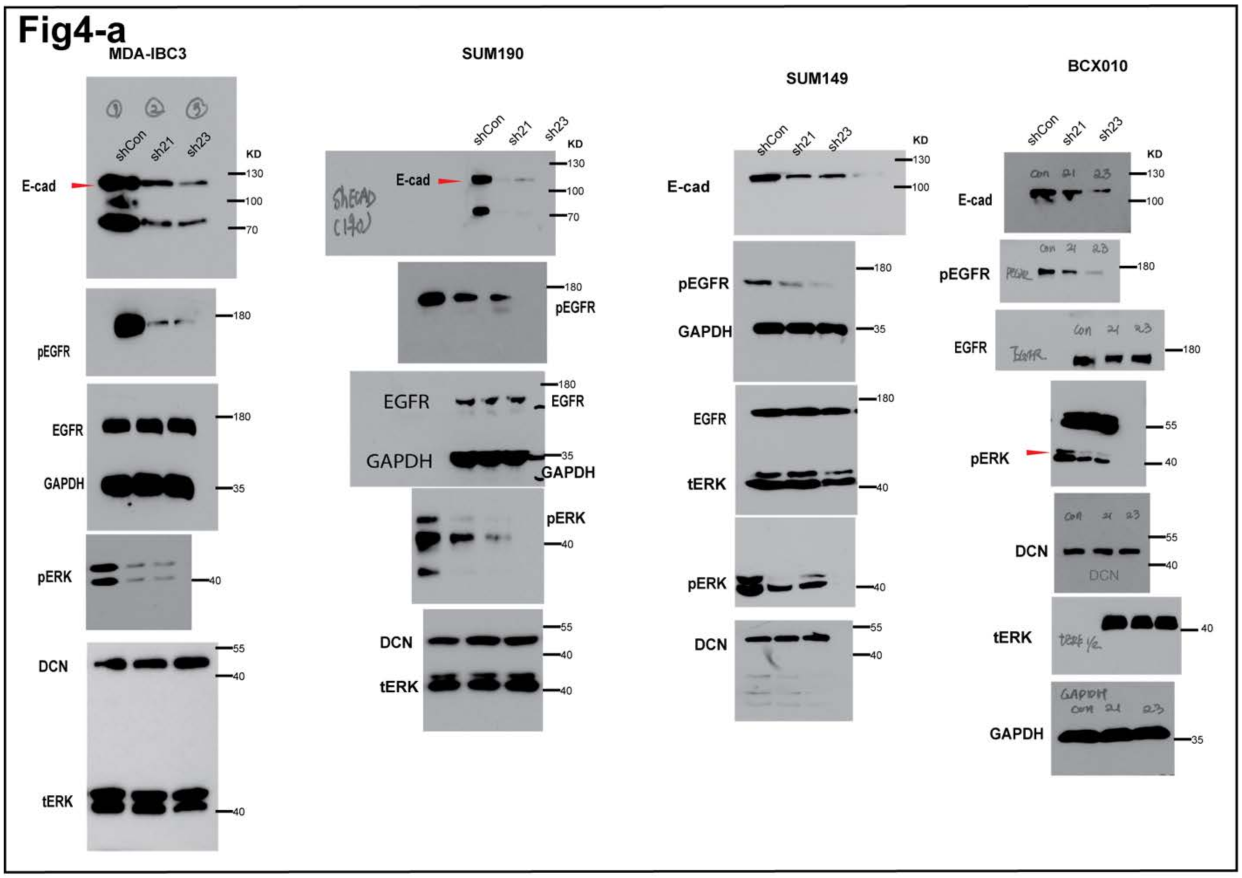
Fig4-a
MDA-IBC3
SUM190
SUM149
BCX010
shCon
sh21
sh23
KD
E-cad
pEGFR
EGFR
GAPDH
pERK
DCN
tERK
130
100
70
180
55
40
35

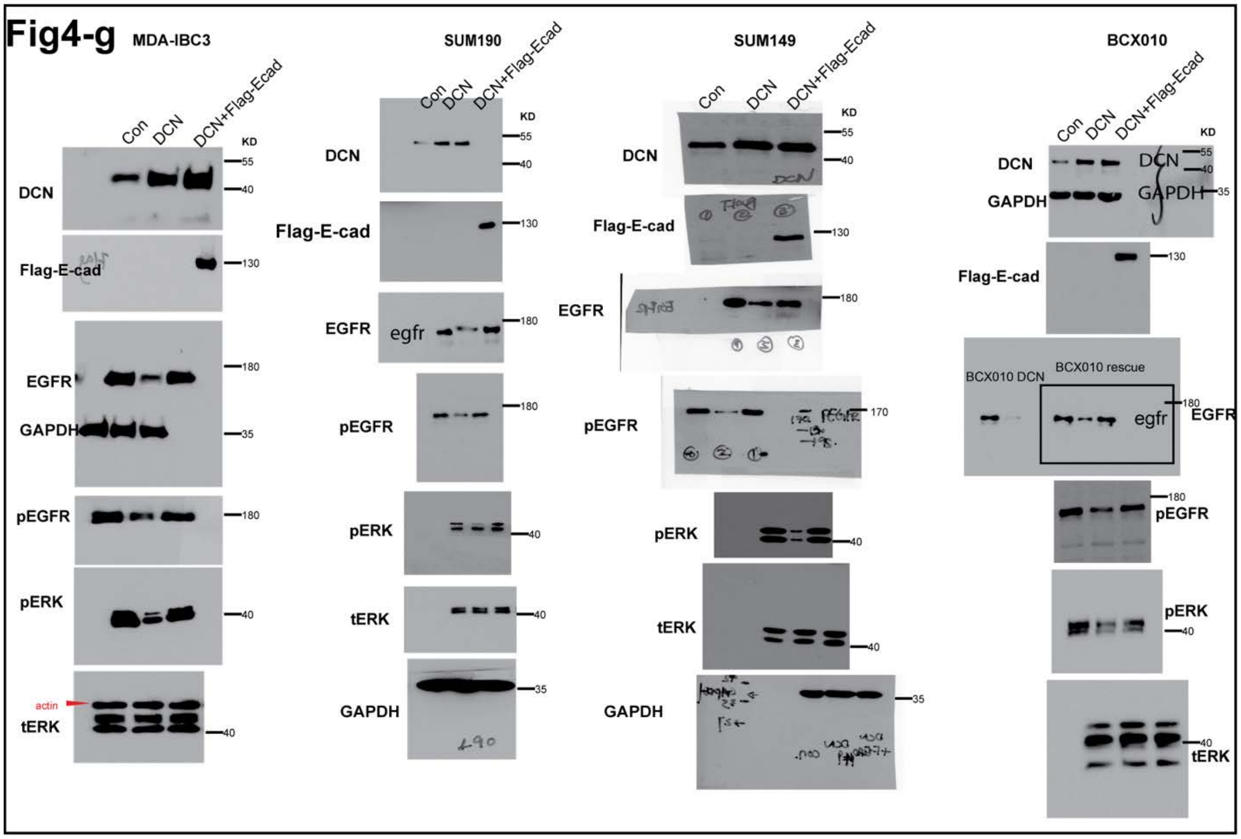
Fig4-g
MDA-IBC3
SUM190
SUM149
BCX010
Con
DCN
DCN+Flag-Ecad
KD
DCN
GAPDH
Flag-E-cad
EGFR
pEGFR
pERK
tERK
actin
BCX010 DCN
BCX010 rescue
55
40
35
130
180
170

Fig5-a

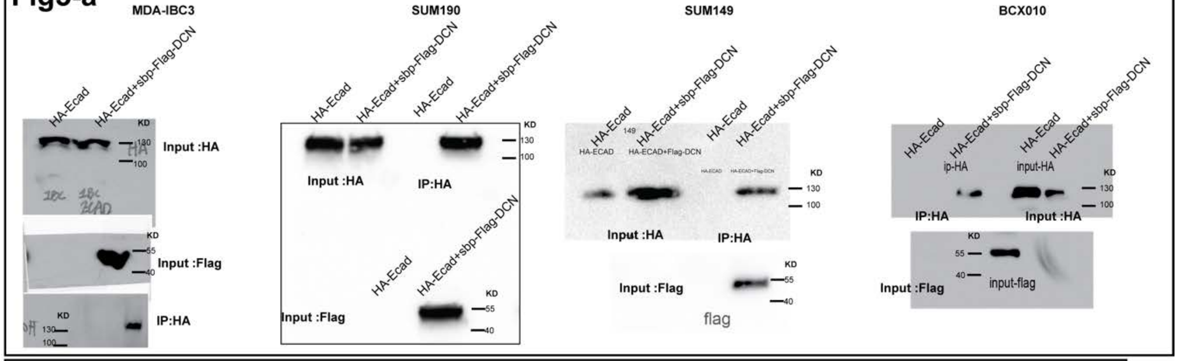

Fig5-b

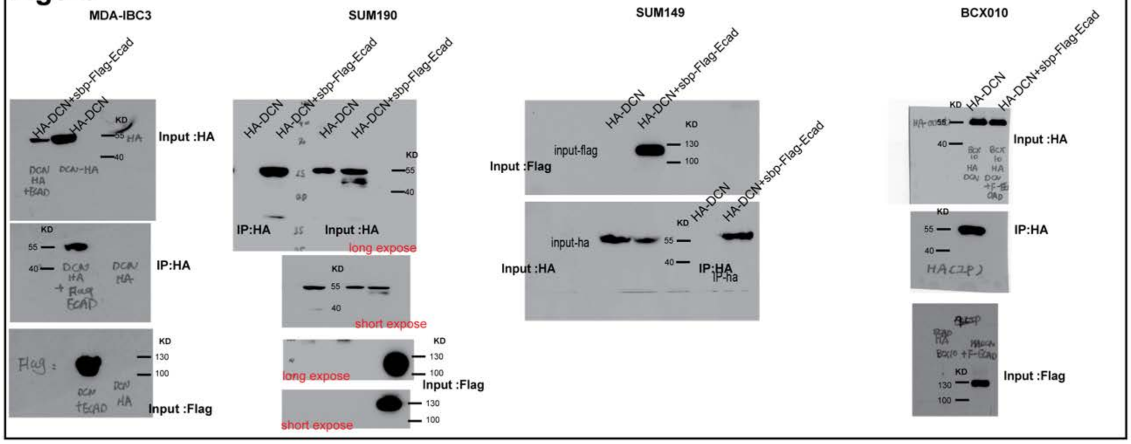

Fig5-c

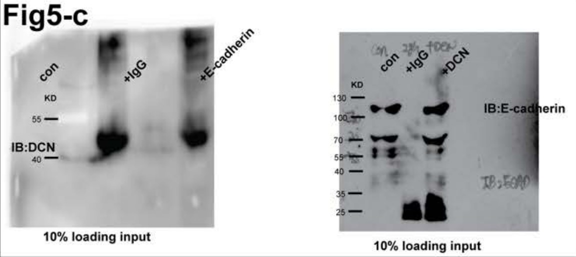

Fig5-d

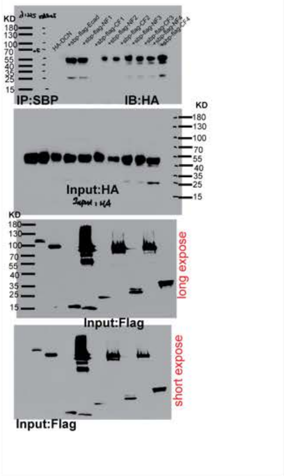

Fig5-e

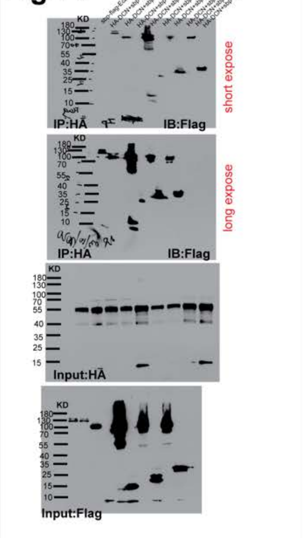

Fig6-a

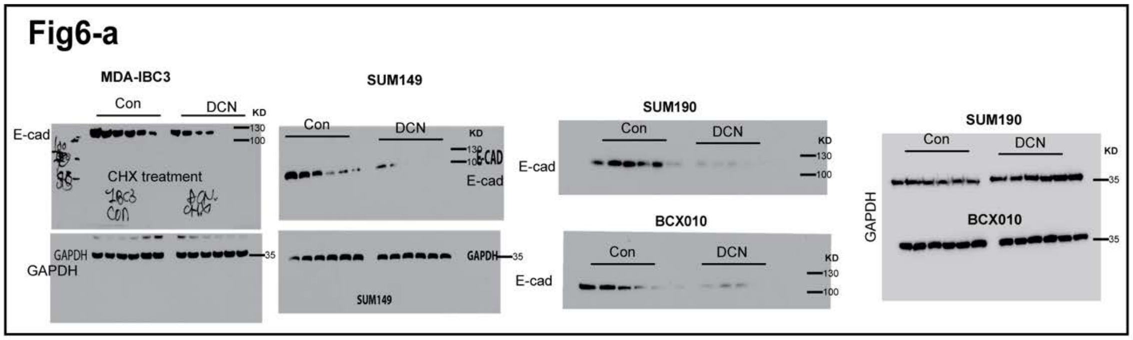

Fig6-b

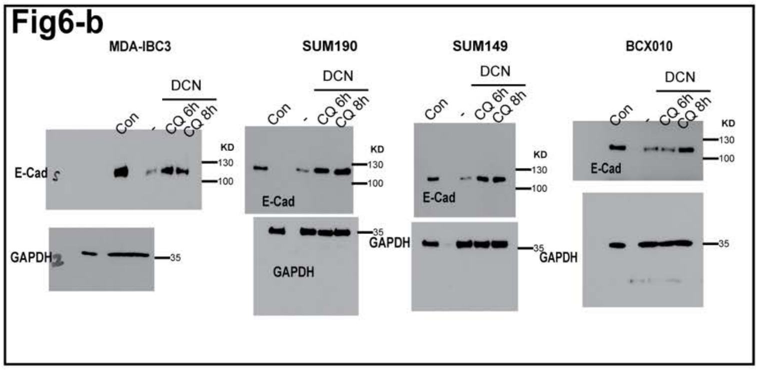

Fig6-c

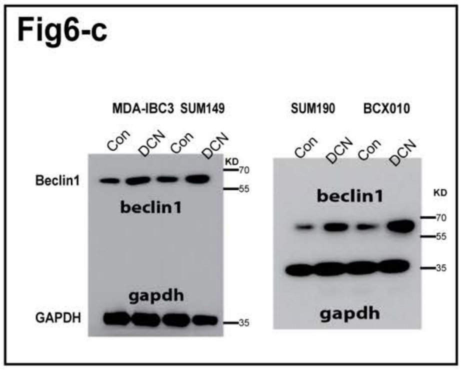

Fig6-d

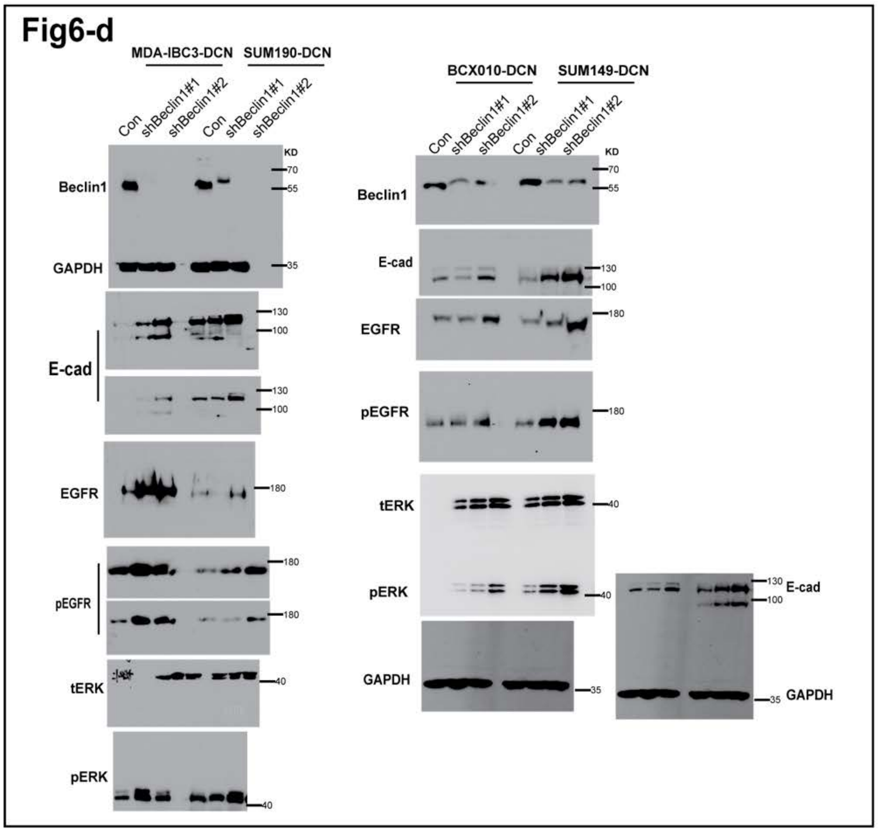

Supplementary Fig 9a

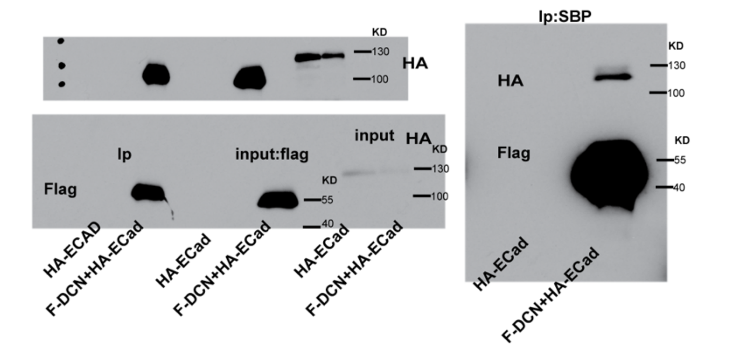

Supplementary Fig 9b

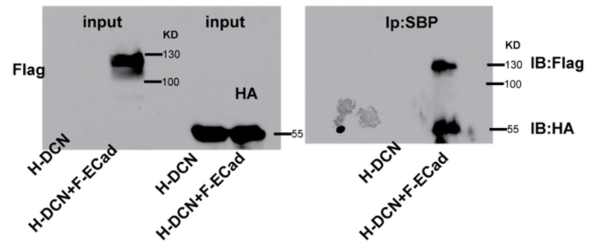

Supplementary Fig12

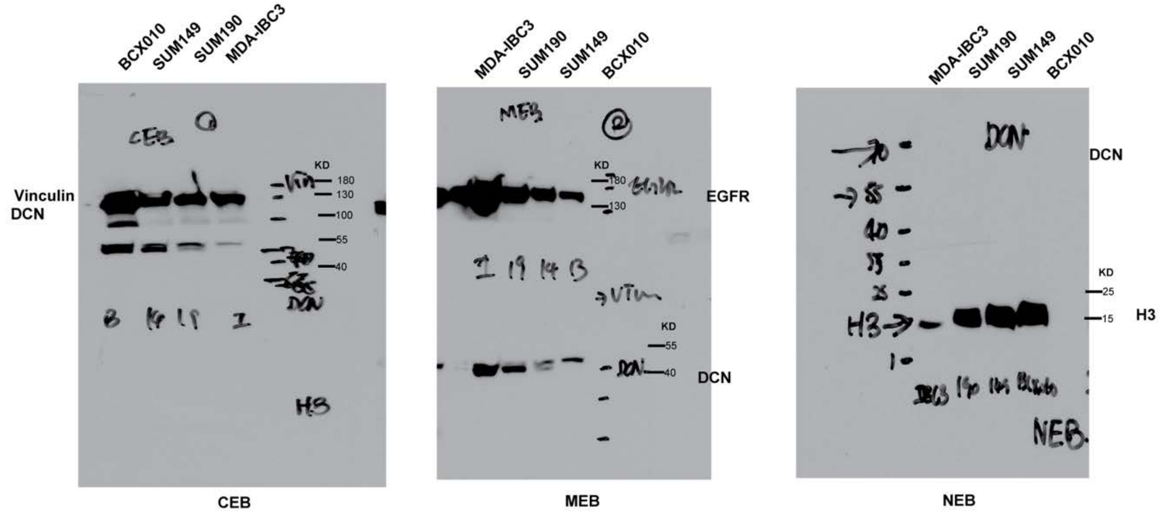

Supplementary Fig 14a

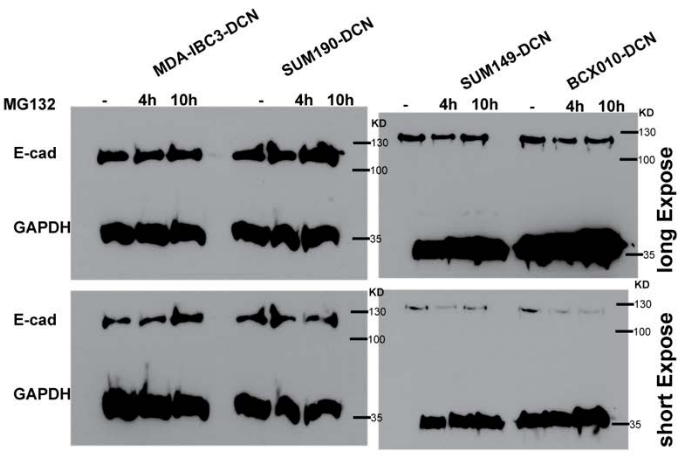

Supplementary Fig 14b

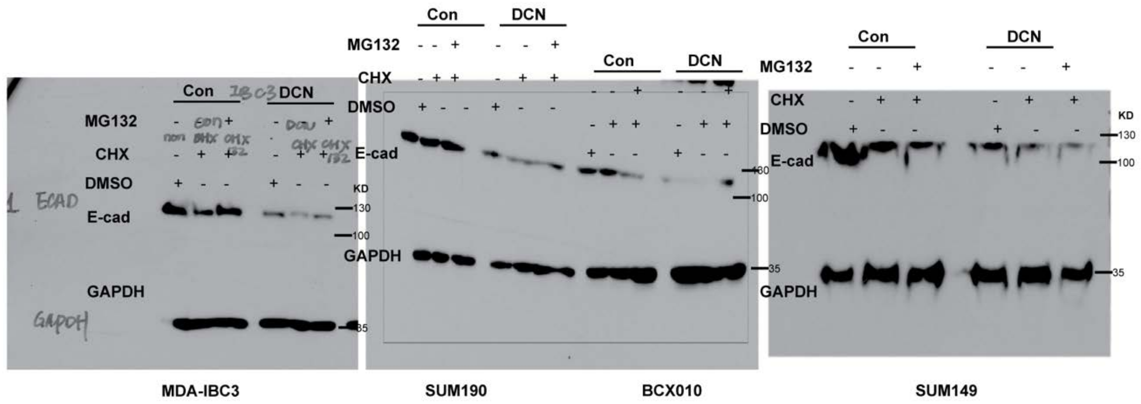

Supplement: Supplementary file 2 — Supplementary Information [file 42003_2020_1590_MOESM2_ESM.pdf]
