# Supplementary material for: Decorin-mediated suppression of tumorigenesis, invasion, and metastasis in inflammatory breast cancer
Source: Commun Biol. 2021 Jan 15;4:72. doi: 10.1038/s42003-020-01590-0 (PMC7811004; doi:10.1038/s42003-020-01590-0)
Supplement: Supplementary file 3 — Description of Additional Supplementary Files [file 42003_2020_1590_MOESM3_ESM.pdf]

Description of Additional Supplementary Files

File Name: Supplementary information
Description: Supplementary Figures with Figure Legends 1-14
Supplementary Figure 15 contains all uncropped western blots for main and Supplementary figures.

File Name: Supplementary Data 1
Description: RPPA (reverse-phase protein array proteomic profiling) results.

File Name: Supplementary Data 2
Description: Sequence details of the oligonucleotides;
The details of antibody dilution;
